# Communication strategies in childhood vaccine hesitancy: a scoping review

**DOI:** 10.11606/s1518-8787.2026060007242

**Published:** 2026-07-17

**Authors:** Elisangela Rodolfo Miras, Cristiane Spadacio, Bruna Aparecida Gonçalves, Elda de Oliveira, Paulo Henrique Nico Monteiro, Luan Nascimento Lázaro, Pamela Machado Polato, Júlia Zaparolli, Lorruan Alves dos Santos, Camila Carvalho de Souza Amorim Matos, Carolina Luisa Alves Barbieri, Marcia Thereza Couto

**Affiliations:** I Universidade de São Paulo. Faculdade de Medicina. Departamento de Medicina Preventiva. São Paulo, SP, Brasil; II Universidade Católica de Santos. Programa de Pós-Graduação em Saúde Coletiva. São Paulo, SP, Brasil; III Instituto Butantan. Laboratório de Ecologia e Evolução. São Paulo, SP, Brasil; IV Universidade Federal de Santa Catarina. Departamento de Ciências Médicas. Araranguá, SC, Brasil; V Fundação Butantan. Centro de Ensaios Clínicos e Farmacovigilância. São Paulo, SP, Brasil

**Keywords:** Vaccination, Vaccination Hesitancy, Health Communication, Scoping Review

## Abstract

**OBJECTIVE:**

To analyze how communicative configurations are addressed in empirical studies on childhood vaccine hesitancy, with an emphasis on the strategies and communication channels used, as well as on how these elements influence parents’ and caregivers’ decisions.

**METHODS:**

A scoping review was conducted across five databases (Scopus, Web of Science, PubMed, SciELO, and LILACS) on childhood vaccine hesitancy and communication strategies, including articles published between 2015 and 2017 in English, Portuguese, Spanish, and French. Empirical studies on childhood vaccination were included, with children’s age as defined by the study authors. Data extracted independently by five researchers were analyzed through thematic synthesis, based on a coding matrix that categorized findings into core themes. The analysis was theoretically guided by Hepp and Hasebrink’s concept of communicative configurations.

**RESULTS:**

Twenty-two studies were analyzed, mostly conducted in the United States (n = 9), primarily involving parents/caregivers and healthcare professionals. Social media emerged as a central medium, with an ambivalent role: as a source of misinformation, but also a channel for pro-vaccine messages. Strategies featuring clear scientific messages, the MOTIVE tool, and analyses of anti-vaccine discourses were promising in increasing vaccination intention. Trust-based relationships between physicians and families and message tailoring were identified as fundamental.

**CONCLUSION:**

Health communication is essential for addressing childhood vaccine hesitancy. The reviewed studies indicate that integrated strategies combining evidence-based public policies with personalized and culturally sensitive communication are key to promoting vaccine uptake and increasing confidence in vaccines.

## INTRODUCTION

Vaccination is recognized as one of the main health strategies for reducing infectious diseases and associated mortality^1^. In Brazil, childhood vaccination is part of a set of actions aimed at individual and collective protection against vaccine-preventable diseases, including the administration of vaccines from birth to 10 years of age, as set out in the National Vaccination Calendar^2^. These actions were consolidated with the creation of the Brazilian National Immunization Program in 1973 and, in the case of children, are supported by the Statute of the Child and Adolescent^3^, which guarantees mandatory vaccination, except in cases of medical contraindication.

Despite its proven effectiveness, vaccination has been impacted by the phenomenon of vaccine hesitancy, a concept formalized in 2014 by the Strategic Advisory Group of Experts on Immunization (SAGE) of the World Health Organization (WHO). Vaccine hesitancy is a complex and context-specific phenomenon, varying according to time, place and type of vaccine. It is influenced by multiple factors, including complacency, convenience and trust, the so-called 3Cs, later expanded to the 5Cs (complacency, confidence, convenience, calculation, and collective responsibility), reflecting the conceptual evolution promoted by SAGE. Initially, the WHO defined vaccine hesitancy as “delay in accepting or refusing vaccines, despite the availability of vaccination services^”^. Before this standardization of terminology, the literature used terms such as “vaccine refusal” and “anti-vaccine” to describe the phenomenon^5^. More recently, the WHO, through the BeSD (Behavioral and Social Drivers of Vaccination) model, proposed a new definition of vaccine hesitancy as “a motivational state of conflict or opposition to vaccination, which includes intentions and willingness^”^, replacing the original 2014 formulation.

Several studies have identified recurring beliefs and fears among parents and caregivers about vaccination: fear of adverse events^7,8^; reduced perception of the risk of the disease^9^; distrust of the pharmaceutical industry^8,9^; questions about the composition of vaccines^10,11^; belief in the superiority of natural immunity over that conferred by vaccination^8,10^ and religious convictions^11^. Moreover, the internet, especially social media, is an important source of disinformation about vaccines, spread mainly, although not exclusively, by anti-vaccine groups^12^.

The Covid-19 pandemic has aggravated vaccine hesitancy and its effects on childhood immunization, the short- and medium-term consequences of which are still little known^13^. Among the impacts observed are the drop in vaccination coverage, the discrediting of vaccines, and the rejection of immunization actions by some segments of the population^14^. The decision to vaccinate, although individual, is socially shaped and crossed by inequalities that influence both vulnerability to illness and access to health services, reinforcing social and health inequalities^5,11^.

In this context, health communication is a strategic tool for tackling vaccine hesitancy^15^. However, studies indicate that the dissemination of scientific data, based on the premise of an information deficit, does not guarantee greater confidence in vaccines or a sustained increase in vaccination coverage. Effective communication strategies for certain groups can be innocuous or counterproductive for others when they ignore previous attitudes and cultural specificities^15,16^, and the absence or weakness of well-structured strategies compromises the effectiveness of immunization actions and favors the mobilization of groups opposed to vaccines^17^.

Given the breadth of the research question, the heterogeneity of empirical studies and the interest in mapping the extent, nature, and gaps on the subject, we opted for a scoping review. The article aims to analyze research on communication strategies aimed at childhood vaccine hesitancy, identifying the media used, the approaches adopted, and the factors that influence the choices of parents and caregivers in vaccination decision-making.

## METHODS

This is a scoping review, conducted according to the methodology of the Joanna Briggs Institute (JBI) and reported according to the PRISMA extension for scoping review (PRISMA-ScR)^18^, to ensure transparency and methodological rigor. Among the steps followed were the definition of eligibility criteria, careful selection of studies by reading titles, abstracts, and full texts, and standardized data extraction, ensuring consistency and reproducibility throughout the process. In the review, the definition of “child” presented in the articles themselves was adopted, and the phenomenon of interest was vaccine hesitancy in this age group.

The following inclusion criteria were established for the eligibility of the studies: research published between 2015 and 2023, a period defined as following the formalization of the concept of vaccine hesitancy by SAGE in 2014, in texts available in English, Spanish, Portuguese, and French; studies that addressed communication strategies related to childhood vaccine hesitancy; investigations that explored means, approaches, or interventions aimed at communication between health professionals, parents or caregivers; and empirical research, regardless of methodological design. Review studies were excluded, as were those that dealt exclusively with adult vaccination or immunization in general.

The information sources selected were: Scopus, Web of Science, PubMed, Scientific Electronic Library Online (SciELO), and Latin American and Caribbean Health Sciences Literature (LILACS). The following DeCS/MeSH descriptors were used to construct the search strategies: Health Communication; Vaccine Hesitation; Vaccine Refusal; Movement Against Vaccination; Communication; Children’s Health. These were used in Portuguese for the searches in the SciELO and LILACS databases. In the other databases, they were used in English. The Boolean operators OR and AND were used to select the articles, considering each information source.

The databases were searched on April 11, 2025. The results were imported into the Rayyan software, where duplicate texts were excluded. Two researchers then independently and blindly screened the titles and abstracts, calling in a third researcher if there were any discrepancies. Twenty-five articles were read in full and three were excluded because they did not address communication strategies related to (non-)vaccination. In the end, 22 articles were included. The process of selecting studies is shown in the diagram based on the PRISMA protocol (Figure).

**Figure:**
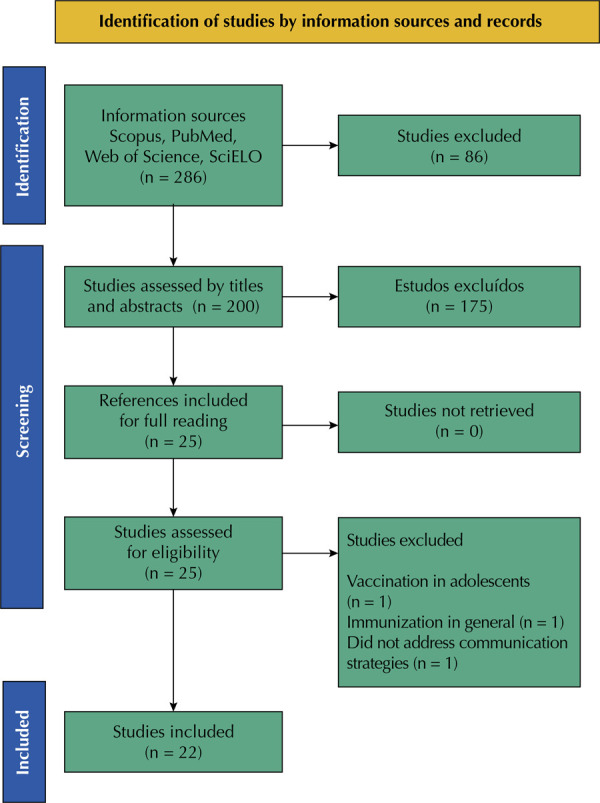
Diagram based on the PRISMA protocol for scoping review (PRISMA-ScR).

Data extraction was carried out independently by five researchers. Any doubts or inconsistencies identified during the process were discussed and resolved in collective meetings. The process took place on the Excel platform and was guided by the framework of communicative configurations in the field of communication^19,20^, applied in this study to health communication.

The extraction process followed four main axes: 1) Characterization of the articles, including identification of the authors, year of publication, title, DOI, journal, institution, country, and gender of the corresponding author, used as a gender proxy. 2) Contextualization of the studies, including the theoretical framework referenced, definition of the object of study, research objectives, geographical location (country, state, region, or *locus*), and groups or populations analyzed. 3) Theoretical framework and methodological delimitation, encompassing the concept of vaccine hesitancy adopted, the media used, the perspectives of the actors involved (parents/caregivers/guardians, health professionals or managers) in relation to vaccination and/or vaccine hesitancy, and the communication strategies employed in the different media. Finally, the fourth axis refers to the synthesis, in which the communication strategies and their outcomes were identified.

To understand the communicational dynamics related to vaccine hesitancy, we adopted the concept of communicative configurations, defined by Hepp and Hasebrink^20^ as “patterns of communicative intertwining processes that exist across various media and have a thematic framework that guides communicative action” (p. 12).

Communicative configurations articulate four interdependent dimensions: (i) forms of communication – the modalities of mediated interaction; (ii) set of media – the platforms that sustain communicative action; (iii) constellation of actors –the subjects who recognize themselves as part of that configuration; and (iv) thematic framework – the central theme that gives meaning to the interaction. These elements are dynamic and shaped by the process of mediatization, a central concept for analyzing the relationship between media transformations, communication practices and socio-cultural changes^19,20^.

Data analysis was guided by the theoretical framework of communicative configurations, as proposed by Hepp and Hasebrink^20^. In the light of this framework, thematic synthesis was used as a strategy for processing the information, based on a coding matrix which made it possible to group the findings into central themes.

This review was not registered on a public platform such as the International Prospective Register of Systematic Reviews (Prospero) or the Open Science Framework (OSF). The data extraction forms and coding matrix used in the thematic synthesis are available on request from the authors.

## RESULTS

Twenty-two articles were included in this review. Chart 1 summarizes the main information from the studies analyzed. As for where the research was carried out, the majority was conducted in the USA (n = 9), followed by Australia (n = 4) and Italy (n = 2); there were also studies conducted on the “internet”, with data obtained in English. Studies were also identified in Brazil, Turkey, Pakistan, India, Scotland, and Poland. The groups investigated were: parents and caregivers, with two studies focusing specifically on mothers; health professionals (pediatricians, family doctors and nurses responsible for vaccination); the general population; adolescents and university students (undergraduate and medical residency).

**Chart 1:** Summary of the characterization of the studies analyzed.

Author	Objective	Methodological design	Country, state, region	Research context	Study groups/populations
Hughes et al.^31^ (2021)	To identify rhetorical strategies in vaccine hesitancy discourse and develop a codebook of the most recurrent anti-vaccination themes, both general and specific to Covid-19	Qualitative – content analysis and codebook production	USA	Social media (Facebook) and websites	Parents/caregivers
Kim et al.^39^ (2022)	To test the effectiveness of messages in changing parents’ attitudes and behavioral intentions about HPV vaccination	Quantitative	USA	Social media (Facebook) and websites	Parents/caregivers living in the USA
Cole et al.^22^ (2022)	To evaluate the impact of an educational intervention and a communication tool based on motivational interviewing on childhood vaccination coverage	Quantitative – in-service intervention	USA	Intervention using the Motive strategy (Motivational Interviewing Tool to Improve Vaccine Acceptance)	Health professionals: pediatricians and general practitioners
Duchsherer et al.^30^ (2020)	To analyze how narrative strategies among parents who are hesitant or opposed to vaccination contribute to building communities among parents who are hesitant and refuse to vaccinate.	Qualitative – thematic analysis	USA - states of: Virginia, West Virginia, Florida, Oregon and Michigan	Parent/caregiver testimonials on The VaxXed website	Parents/caregivers
Silva et al.^17^ (2023)	To evaluate the effect of different communication strategies on the intention to vaccinate oneself and one’s children, considering previous attitudes of vaccine hesitancy.	Quantitative – communication intervention	Brazil	Simulated environment (online)	People over 18 living in Brazil
Llavona-Ortiz et al.^40^ (2022)	To characterize the type of information about the HPV vaccine viewed on social networks, parents’ engagement with this content and its relationship with vaccination decisions.	Quantitative	USA	Social media	Parents/caregivers
Hopfer et al.^38^ (2019)	To characterize HPV vaccine recommendation patterns among pediatricians, identifying communication profiles associated with parental hesitancy.	Quantitative	USA	Context of health services	Parents/caregivers
Özen et al.^33^ (2022)	To explore family physicians’ experiences with antivaccine parents and provide data on the antivaccination movement in Turkey.	Qualitative – phenomenological study	Turkey - Sakarya province	Context of health services	Health professionals - family doctors
Akram et al.^36^ (2021)	To examine the influence of communication channels, especially electronic and social media, on parental decision about typhoid vaccination.	Quantitative	Pakistan - Karachi city	Different media, including social media	Parents/caregivers
Glanternik et al.^23^ (2020)	To evaluate the impact of a communication tool for doctors on comfort and perceived effectiveness when dialoguing with hesitant parents.	Quantitative – in-service intervention	Internet	Integrated healthcare delivery system	Health professionals - pediatricians and family doctors
Steffens et al.^25^ (2021)	To compare the effectiveness of different strategies for debunking myths about vaccines on parental intention to vaccinate their children.	Quantitative – communication intervention	Australia	Simulated environment (online)	Parents/caregivers
Barton et al.^21^ (2022)	To verify whether training residents in a structured communication method influences behaviors when interacting with hesitant parents.	Quantitative – educational intervention	USA –University of Louisville	Simulated environment (online)	Pediatric residents
Randall et al.^34^ (2020)	To identify communication strategies and challenges used by experienced professionals in conversations with highly hesitant parents and propose ways to improve clinical practice.	Qualitative – thematic analysis	Australia – Melbourne and Sydney	Context of health services	Health professionals
Brunelli et al.^37^ (2021)	To assess the reach of a revaccination campaign after vaccine failure and to investigate levels of parental trust in vaccines and sources of information.	Quantitative	Italy – town of Codroipo	Context of health services	Parents/caregivers
Panozzo et al.^41^ (2020)	To compare the impact of grouped recommendations and personalized messages about maternal concerns on the intention to vaccinate against HPV.	Quantitative – randomized clinical trial	USA	Simulated environment (online)	Parents/caregivers
Berry et al.^29^ (2018)	To identify parents’ communication needs about immunization to support the development of the Sharing Knowledge About Immunization/SKAI support system.	Qualitative – grounded theory	Australia	Two large cities and one regional catchment area with a high prevalence of vaccine objection	Parents/carers
Shay et al.^34^ (2018)	To describe forms of expression of vaccine hesitancy among parents, professional responses and associations with same-day vaccination.	Qualitative – content analysis	USA – Dallas, Texas	Context of health services	Parents/caregivers, adolescents and health professionals
Berry et al.^28^ (2017)	To explore the experiences of primary care professionals in consultations with parents who refuse vaccines, as well as the challenges and management strategies.	Qualitative – grounded theory	Australia	Context of health services	Health professionals
Nagar et al.^24^ (2018)	To evaluate the adoption and impact of the Khushi Baby digital system on child vaccination adherence in rural communities.	Quantitative –community intervention	India	Rural regions of Udaipur, Rajasthan	Parents/caregivers
Pluviano et al.^25^ (2017)	To compare the effectiveness of three strategies for correcting misinformation about vaccines in a controlled experimental setting.	Intervention study	Italy – University of Naples and Scotland - University of Edinburgh	Simulated environment (online)	Students from these two universities
Lockhart et al.^32^ (2018)	To understand how a multifaceted communication intervention applied by medical teams can improve HPV vaccination rates.	Qualitative - process evaluation	USA - Denver, Colorado.	Context of health services	Parents/caregivers and health professionals
Dolinski et al.^27^ (2023)	To test the effectiveness of the “But You Are Free” (BYAF) technique on newborn vaccination adherence.	Experimental design	Poland	Context of health services	Parents/caregivers
Seytre et al.^42^ (2024)	To develop an evidence-based communication strategy to increase vaccination coverage in Niger.	Mixed methods	Niger	General population	General population, including adults responsible for children

HPV: human papillomavirus.

In terms of methodology, the study designs were varied: quantitative intervention studies^17,21^, including one with an experimental design^27^; qualitative studies^28^; quantitative studies^36^; and one using mixed methods^42^. The studies analyzed different perspectives on childhood vaccine hesitancy. One set looked at the rhetoric built up around the phenomenon^30,31,33,35^. Others focused on communication between parents/caregivers and/or health professionals in social networks^31,39,40^. There have also been studies on trust in vaccines and the influence of the anti-vaccine movement on parents and caregivers^33,37^.

Several studies have discussed communication strategies in the relationship between health professionals and parents/caregivers^17,23,26,29,34^. Other studies have focused on communication strategies aimed at reducing vaccine hesitancy^17,22,24^, as well as specific studies on reducing vaccine hesitancy^25,26,41^.

The thematic synthesis carried out took into account the four central analytical aspects established on the basis of Hepp and Hesebrink’s theoretical framework^20^, namely: 1) The concept of vaccine hesitancy; 2) The communication perspective used: who it is aimed at and how it is produced; 3) The types of communication strategies discussed; and 4) Anti-vaccine communication strategies (Chart 2).

**Chart 2:** Thematic summary of the findings according to Hepp and Hasebrink’s four analytical aspects20.

Analytical aspect	Themes	Summary of findings
1. Concept of vaccine hesitancy	1.1 Classic definition (WHO/SAGE)	The WHO/SAGE definition predominated, which understands vaccine hesitancy as delaying or refusing vaccination despite the availability of services^17,21–25,31^.
	1.2 Conceptual broadening: complex social phenomenon	Some studies broaden the concept, understanding vaccine hesitancy as a complex social phenomenon, including the notion of “state of mind”, a phenomenon in continuum, cognitive mechanisms and misinformation, as well as historical-political perspectives^21,22,25,30,35^.
	1.3 Lack of explicit definition	Some studies do not present a formal definition, associating hesitation mainly with misinformation^26,36,40^, institutional distrust^29,42^, or religious factors^33^. Studies with no explicit definition^27,29,32,38,39,41^.
2. The communicational perspective: who is it aimed at and how is it produced?	2.1 Centrality of parents and caregivers	Most studies address parents and caregivers, especially in the context of childhood vaccine hesitancy^24,26–30,32,35–41^. Some of the literature focuses specifically on parents and caregivers identified as hesitant^29,33,38,41^.
	2.2 Health professionals as a target audience	Some studies have analyzed strategies aimed at health professionals alone^22,29,33,34^, or together with parents/caregivers^24,28^.
	2.3 General population and digital environments	Few studies address the general population^17,31,37^ or analyze social networks, websites and digital platforms (Facebook/Instagram) as central spaces for communication production^22,31,40^.
3. Types of communication strategies discussed	3.1 Mapping narratives and anti-vaccination rhetoric	Studies that prioritize rhetorical mapping and the identification of antivaccine narratives, with the construction of analytical codebooks^31^.
	3.2 Testing messages and communication frameworks	Testing the use of messages according to thematic axes and informational and narrative frameworks^39^. As well as approaches based on Effective Communication without Confrontation centered on listening, validation and dialogue^23^.
	3.3 Limits of traditional corrective strategies	Strategies based on “myths versus facts”, questions or statements show limited results or no significant differences, with indications of a possible rebound effect^25,26^.
4. Anti-vaccine communication strategies	4.1 Digital ecosystems and the dispute over meanings	Social networks are heterogeneous ecosystems, marked by disputes over meanings and the circulation of antivaccine narratives based on antagonism and harm^31^. Online communities mobilize identity belonging, articulating discourses of mistrust, collective identity and a sense of belonging based on this theme^30^.
	4.2 Infodemic and amplification of mistrust	Rumors and the infodemic contribute to increasing community distrust and weakening institutional trust^42^.
	4.3 Evidence-based counterpoint strategies	Messages based on scientific evidence increase vaccine intention^39^, and structured interventions such as Motive can support professionals and increase vaccination coverage^22^.

SAGE: Strategic Advisory Group of Experts on Immunization; WHO: World Health Organization.

Regardim item 1 (concept of vaccine hesitancy), some studies have adopted the 2014 WHO definition^4^ which considers vaccine hesitancy to be a delay in accepting or refusing a vaccine, despite its availability^17,21,23^. Among these, three studies define vaccine hesitancy as a complex social phenomenon, related to beliefs about health in its individual, sociocultural and political dimensions^21,22,25^. Other studies advance the definition, treating vaccine hesitancy as a “state of mind” permeated by uncertainties, conflicts or oppositions^21^, or as part of a *continuum* of indecision^35^, or even as a set of cognitive mechanisms that favor misinformation^25^. One study presented an expanded historical perspective for the phenomenon, in which vaccine hesitancy is rooted in emotional, social, cultural and political aspects that make misinformation “sticky” and belief in vaccines counterintuitive^30^.

It is worth noting that some studies did not provide an explicit definition of the concept of vaccine hesitancy^27,29,32,35,39,41^, but linked it to misinformation^26,36,40^, mistrust of vaccines due to their potential side effects^29^, or religious motivations^33^.

As for the target audience of the communication, which relates to analytical aspect 2. The communication perspective used: who it is aimed at and how it is produced, 13 studies focused on parents and/or caregivers^22,24,26,27,29,30,35,36,39^, four specifically addressed hesitant parents/caregivers^29,32^. Eight studies focused on health professionals^21^. Two studies considered both parents/carers and health professionals^28,32^ and three focused on the general population^17,25,31^.

With regard to the set of media used to frame the topic, four studies more specifically highlighted content produced on social platforms such as Facebook and Instagram^17,22,31,39^. Only two of the 22 studies analyzed social networks (Facebook) and websites as sources of information^31,39^. As for analytical aspect 3 (types of communication strategies discussed), one study analyzed anti-vaccine discourses from a rhetorical approach and the context of their production, resulting in the production of a book with narrative codes recurrently used in online anti-vaccination messages, with the aim of helping different actors to confront misinformation during the Covid-19 vaccination campaign^31^. Kim et al.^39^ developed messages structured around five themes: (1) concerns about the safety and side effects of vaccines; (2) distrust in the health system; (3) doubts about the effectiveness of the vaccine; (4) associations with sexual activity; and (5) misinformation about HPV and its vaccine. The communication strategy “Effective Communication Without Confrontation” (ECC) has proved to be effective in establishing dialogues between doctors who attend hesitant families, contributing to the establishment of constructive dialogues^23^.

Other communicative approaches, such as the use of myths, questions and affirmations, were also tested, but showed no significant differences in the intention to vaccinate^39^. Corrective strategies of the “myth *versus* fact*”* type *can* have a rebound effect by reinforcing false information, such as the association between vaccines and autism^37^.

In analytical aspect 4 (anti-vaccine communication strategies), specifically on social networks, there was a wide and heterogeneous range of activities, from government authorities and experts to the press and the general public^31^. Some narratives construct antagonists as a “dark villain” or present the vaccine itself as a source of harm, especially regarding vaccination against Covid-19. In contrast, experimental interventions with parents have shown that evidence-based messages increase the intention to vaccinate children against HPV^39^, and that tools such as Motive can support health professionals and favor vaccination coverage^22^.

Studies have shown that exposure to anti-vaccine discourse, especially about HPV, can lead to refusal or delay in immunization^40^. The recommendation of this vaccine is influenced by parental perception of the risk of anticipating sexual life, generating ambivalence among health professionals^38^. Testimonials from parents on the VaxXed website reveal distrust of doctors, self-diagnosis, defense of freedom of choice and building a sense of community, expressing the complex relationship between information and trust in vaccines^30^. Rumors about Covid-19 vaccines, intensified by the infodemic, have also aroused distrust in the community, although health professionals maintain a high degree of confidence^42^.

Building bonds between doctors and parents/caregivers has been shown to be essential in alleviating concerns and increasing opportunities for vaccine recommendations^34^. In anti-vaccine families, doctors clarified doubts about side effects, the origin of vaccines and religious objections. The study by Özen et al.^33^ showed that doctors adopted a non-judgmental attitude, listened attentively to parents and provided information about immunizers and the risks of not vaccinating. More experienced doctors reported using their own clinical cases as a persuasive resource. Although they did not impose vaccination, they sought to convince parents, achieving success, supported by trust, effective communication and the sharing of scientific evidence^33^. Interventions that addressed specific concerns increased the intention to vaccinate^41^.

A comparison between groups of hesitant and less hesitant parents revealed that the greater the vaccine hesitancy, the greater the demand for transparency and information^29^. Specific interventions, such as information sheets, images of diseases and presumptive approaches, have been shown to be positive for HPV vaccination adherence, receiving approval from professionals and parents^32^. On the other hand, the BYAF technique (But-You-Are-Free Technic), which can discourage rather than encourage vaccination, should be used with caution^27^.

## DISCUSSION

The thematic framework of vaccine hesitancy refers to the concept presented by the conceptual model developed by SAGE, aimed at understanding the factors that influence the acceptance or refusal of vaccines. Even so, the articles analyzed reveal diversity in the way the phenomenon is understood; some provide explicit definitions, such as the one proposed by the WHO, while others have no conceptualization.

The WHO references include communication aspects among the factors that influence vaccine hesitancy. In addition, the documents highlight that communication is essential to the success of immunization programs and, when poorly used, can increase vaccine hesitancy^4,6^.

Vaccine hesitancy involves uncertainty, indecision, conflict or opposition to vaccination^11^, going beyond simple refusal or delay. It is a phenomenon that is distributed along a *continuum*, in which many individuals do not identify themselves as “pro-vaccine” or “anti-vaccine”, and it becomes particularly urgent when it is expressed as total refusal or delays of some or all vaccines^10^.

Klintman^43^ divides vaccine hesitancy into two types: irrational contumacy and contumacy motivated by lack of knowledge. In the first case, the attitude is based on a “more archaic rationality”, linked to the body’s defense mechanisms against invisible threats. In the second case, greater and better access to information can make a positive difference in segments of the population whose vaccine hesitancy is moderate or low.

The use of broad definitions or the lack of conceptual clarity makes it difficult to compare results, operationalize data and reach consensus among researchers^5,44,45^. Considering the complexity of the phenomenon, Couto^46^ defends the need for a critical reflection on the traditional, often negative view of individuals or groups who resist vaccination, recommending an approach that takes into account multiple facets, including social aspects and health practices.

Refusal to vaccinate, as distinct from vaccine hesitancy, is a multifaceted phenomenon, with different motivations^47^. Parents identified as “refusers” are not a homogeneous group: they may reject all vaccines or only some, due to concerns about efficacy, adverse effects, distrust of public policies or the pharmaceutical industry. As Sobo points out^47^, there is a difference between refusing due to lack of access and deliberately refusing. Taking inspiration from Marcel Mauss, MacGranahan^48^ proposes understanding refusal as a socially dense gesture, rooted in historical, ethical and cultural contexts. Refusing to vaccinate can operate as an ethical stance that redefines hierarchies and affirms other ways of living and caring^48^.

Refusal manifests itself as an active stance that rejects certain afflictions and builds new forms of sociability. More than simple opposition to the state or science, it can represent moral and political commitment, underpinned by convictions about responsible childcare. As McGranahan argues^48^, refusal is more about insistence than resistance: it is an affirmation of values and alternative ways of life. In Sobo’s study^47^, the decision not to vaccinate consolidates social ties between parents who share an individualized and critical view of health.

Based on the empirical studies analyzed and the theoretical-conceptual literature, it is noteworthy that there is no consensus on the uses of the term “vaccine hesitancy”, which may have an impact on the formulation of communication strategies. It can also be seen that most studies focus on the family sphere, especially parents and caregivers, with social networks as the main means of circulating information. We share, with Hepp and Hasebrink^20^, the understanding of communication as a form of social interaction, essential to relationships and symbolic constructions. In this sense, understanding how communication circulates and acquires meaning in society becomes fundamental, which makes the concept of mediatization relevant.

The concept of mediatization describes how the media transform social relations and everyday practices. Two main approaches guide its study: the institutionalist, centered on mass media, and the socio-constructivist, focused on daily communications and the relationship between media evolution and socio-cultural changes^20^. Hjarvard^49^ proposes that the two are complementary, defining mediatization as the process by which social and cultural activities come to be shaped by and dependent on the media - both through the use that individuals and institutions make of it and through the increasing mediation of interactions by specific platforms.

Hepp and Hasebrink^20^ propose using the concept of mediatization to analyze the relationship between transformations in the media, communication and socio-cultural changes. The focus is on understanding how the media shape communication processes and symbolic interactions. In the articles analyzed, the presence of content about vaccination on networks such as Instagram, Facebook and TikTok stood out, characterizing what the authors define as “virtualized media communication”, sustained by intentionally developed interactive systems.

The empirical studies analyzed showed the formulation of various arguments used to support the anti-vaccine discourse on social networks, with an emphasis on the communicative configurations involved in different contexts of child vaccination. These findings converge with the results of a previous systematic review^50^, centered on the Covid-19 pandemic, which identified that beliefs in conspiracy theories, combined with distrust in the benefits of vaccines, government policies, health systems, vaccine developers and service providers, as well as a lack of health information, contribute to the dissemination and consolidation of anti-vaccine discourses. This scoping review, however, broadens this perspective by considering not only the circulation of disinformation, but also the ways in which different media and actors produce, reproduce and dispute meanings about childhood vaccination in the field of health communication, including innovative and effective approaches.

The results indicate that the most effective communication strategies are those that prioritize building trust, establishing an empathetic dialogue and personalizing messages according to the specific concerns of parents and caregivers. Tools such as Motive and approaches such as ECC have shown potential for increasing vaccine acceptance. In turn, evidence-based interventions adapted to the needs of local contexts have shown potential to reduce vaccine hesitancy. On the other hand, information strategies in the “myth *versus* fact” format can have counterproductive effects, reinforcing mistaken beliefs.

These findings reinforce the need to systematically integrate the field of health communication into immunization policies and programs. Incorporating evidence-based communication strategies, such as training health professionals in skilled listening and motivational interviewing, using messages adapted to sociocultural contexts and building networks of trust between professionals, families and institutions, can improve the effectiveness of interventions. In addition, it is recommended that national and local immunization guidelines include specific parameters on risk communication and tackling misinformation as key components for reducing vaccine hesitancy.

Among the limitations of the studies included in the review, we highlight the predominance of exploratory and small studies, mostly conducted in high-income countries, which restricts the discussion of the findings to middle- and low-income contexts.

This scoping review did not include a formal assessment of the risk of bias, which restricts the assessment of the robustness of the findings and prevents firmer inferences about the comparative effectiveness of the communication strategies identified. This decision is in line with the JBI guidelines^18^, according to which scoping reviews do not require mandatory critical appraisal, do not exclude studies based on methodological quality and aim, above all, to map the extent, variety and nature of the available evidence, rather than judging its methodological rigor. Even so, as the empirical literature on the subject advances, it is recommended that future studies, especially those aimed at evaluating the effectiveness of communication strategies to tackle vaccine hesitancy, incorporate a systematic evaluation of the risk of bias, to strengthen the interpretation of effects and comparability between interventions.

## CONCLUSION

The results indicate that the media play an ambivalent role in communicating about vaccines: on the one hand, they convey misinformation and reinforce mistaken beliefs, and on the other, they can disseminate pro-vaccination messages based on evidence and sensitive to the socio-cultural context. Clear and accessible language, the use of tools such as Motive and analysis of anti-vaccine discourse can increase the intention to vaccinate. However, although non-confrontational communication strengthens the doctor-patient relationship, it does not in itself guarantee greater vaccination coverage.

Given these findings, it can be concluded that tackling vaccine hesitancy requires integrated strategies that combine evidence-based public policies and personalized health communication actions that are sensitive to the sociocultural specificities of the public. Strengthening evidence-based communication approaches, combined with socially inclusive immunization policies, can support professional practices and the formulation of guidelines capable of promoting greater confidence in vaccines and sustained adherence to childhood immunization programs.

## Data Availability

Data is available on request from the corresponding author.
