# Supplementary material for: Communication strategies in childhood vaccine hesitancy: a scoping review
Source: Rev Saude Publica. 2026 Jul 17;60:e26. doi: 10.11606/s1518-8787.2026060007242 (PMC13367143; doi:10.11606/s1518-8787.2026060007242)
Supplement: Translation

# Translation: Estratégias comunicacionais em hesitação vacinal infantil: revisão de escopo

**Authors:** Elisangela Rodolfo Miras, Cristiane Spadacio, Bruna Aparecida Gonçalves, Elda de Oliveira, Paulo Henrique Nico Monteiro, Luan Nascimento Lázaro, Pamela Machado Polato, Júlia Zaparolli, Lorruan Alves dos Santos, Camila Carvalho de Souza Amorim Matos, Carolina Luisa Alves Barbieri, Marcia Thereza Couto

**Affiliations:** I Universidade de São Paulo. Faculdade de Medicina. Departamento de Medicina Preventiva. São Paulo, SP, Brasil; II Universidade Católica de Santos. Programa de Pós-Graduação em Saúde Coletiva. São Paulo, SP, Brasil; III Instituto Butantan. Laboratório de Ecologia e Evolução. São Paulo, SP, Brasil; IV Universidade Federal de Santa Catarina. Departamento de Ciências Médicas. Araranguá, SC, Brasil; V Fundação Butantan. Centro de Ensaios Clínicos e Farmacovigilância. São Paulo, SP, Brasil

**Keywords:** Vacinação, Hesitação Vacinal, Comunicação em Saúde, Revisão de Escopo

## Abstract

**OBJETIVO:**

Analisar como as configurações comunicativas são abordadas nos estudos empíricos sobre a hesitação vacinal infantil, com ênfase nas estratégias e meios comunicacionais utilizados, bem como nas formas pelas quais esses elementos influenciam as decisões de pais e cuidadores.

**MÉTODOS:**

Revisão de escopo, conduzida em cinco bases de dados (Scopus, *Web of Science*, PubMed, SciELO e Lilacs), sobre hesitação vacinal infantil e estratégias comunicacionais, com artigos publicados entre 2015 e 2017, nos idiomas inglês, português, espanhol e francês. Foram incluídos estudos empíricos sobre vacinação infantil, com a idade das crianças determinada pelo próprio autor do estudo. Para a análise das informações extraídas por cinco pesquisadores de forma independente, foi realizada síntese temática, com base em uma matriz de codificação, que categorizou os achados em temas centrais. A análise foi orientada teoricamente com base no conceito de configurações comunicativas de Hepp e Hasebrink.

**RESULTADOS:**

Foram analisados 22 estudos, os quais foram majoritariamente realizados nos Estados Unidos (n = 9), envolvendo principalmente pais/cuidadores e profissionais de saúde. As redes sociais destacaram-se como mídia central, com papel ambivalente: como meio de desinformação, mas também de mensagens pró-vacina. Estratégias com mensagens científicas claras, a ferramenta Motive e a análise de discursos antivacina mostraram-se promissoras para aumentar a intenção de vacinar. Relações de confiança entre médicos e famílias e a personalização das mensagens foram identificadas como fundamentais.

**CONCLUSÃO:**

A comunicação em saúde é fundamental no enfrentamento da hesitação vacinal infantil. Os estudos avaliados indicam que estratégias integradas, combinando políticas públicas baseadas em evidências com comunicação personalizada e culturalmente sensível são essenciais para promover adesão à vacinação e maior confiança nas vacinas.

## INTRODUÇÃO

A vacinação é reconhecida como uma das principais estratégias sanitárias para a redução de doenças infecciosas e da mortalidade a elas associada^1^. No Brasil, a vacinação infantil integra um conjunto de ações voltadas à proteção individual e coletiva contra doenças imunopreveníveis, incluindo a administração de vacinas desde o nascimento até os 10 anos de idade, conforme previsto no Calendário Nacional de Vacinação^2^. Essas ações foram consolidadas a partir da criação do Programa Nacional de Imunizações, em 1973, e, no caso das crianças, são respaldadas pelo Estatuto da Criança e do Adolescente^3^, que garante a obrigatoriedade da vacinação, salvo em caso de contraindicação médica.

Apesar de sua eficácia comprovada, a vacinação tem sido impactada pelo fenômeno da hesitação vacinal, conceito formalizado em 2014 pelo *Strategic Advisory Group of Experts on Immunization* (SAGE) da Organização Mundial da Saúde (OMS). A hesitação vacinal é um fenômeno complexo e específico ao contexto, variando de acordo com o tempo, o local e o tipo de vacina. É influenciada por múltiplos fatores, entre eles complacência, conveniência e confiança, os chamados 3Cs, posteriormente ampliados para os 5Cs (complacência, confiança, conveniência, cálculo e responsabilidade coletiva), refletindo a evolução conceitual promovida pelo SAGE. Inicialmente, a OMS definiu a hesitação vacinal como “atraso na aceitação ou recusa de vacinas, apesar da disponibilidade de serviços de vacinação”^4^. Antes dessa padronização terminológica, a literatura utilizava termos como “recusa vacinal” e “antivacina” para descrever o fenômeno^5^. Mais recentemente, a OMS, por meio do modelo BeSD (*Behavioural and Social Drivers of Vaccination*), propôs uma nova definição de hesitação vacinal como “um estado motivacional de conflito ou oposição à vacinação, que inclui intenções e disposição”^6^, substituindo a formulação original de 2014.

Diversos estudos identificam crenças e receios recorrentes entre pais e cuidadores diante da vacinação: medo de eventos adversos^7,8^; percepção reduzida do risco da doença^9^; desconfiança em relação à indústria farmacêutica^8,9^; questionamentos sobre a composição das vacinas^10,11^; crença na superioridade da imunidade natural sobre a conferida pela vacinação^8,10^ e convicções religiosas^11^. Além disso, a internet, especialmente as mídias sociais, constitui importante fonte de desinformação sobre vacinas, difundida principalmente, embora não exclusivamente, por grupos antivacina^12^.

A pandemia de covid-19 agravou a hesitação vacinal e seus efeitos sobre a imunização infantil, cujas consequências, em curto e médio prazos, ainda são pouco conhecidas^13^. Entre os impactos observados estão a queda nas coberturas vacinais, o descrédito em relação às vacinas e a rejeição às ações de imunização por parte de alguns segmentos da população^14^. A decisão de vacinar, embora individual, é socialmente moldada e atravessada por desigualdades que influenciam tanto a vulnerabilidade ao adoecimento quanto o acesso aos serviços de saúde, reforçando as inequidades sociais e em saúde^5,11^.

Nesse contexto, a comunicação em saúde constitui uma ferramenta estratégica para enfrentar a hesitação vacinal^15^. No entanto, estudos indicam que a divulgação de dados científicos, baseada na premissa de déficit de informação, não garante maior confiança nas vacinas nem aumento sustentado da cobertura vacinal. Estratégias comunicacionais eficazes para determinados grupos podem ser inócuas ou contraproducentes para outros quando se ignoram atitudes prévias e especificidades culturais^15,16^, e a ausência ou fragilidade de estratégias bem estruturadas compromete a efetividade das ações de imunização e favorece a mobilização de grupos contrários às vacinas^17^.

Diante da amplitude da questão de pesquisa, da heterogeneidade dos estudos empíricos e do interesse em mapear a extensão, a natureza e as lacunas sobre o tema, optou-se por uma revisão de escopo. O artigo objetiva analisar as pesquisas sobre as estratégias comunicacionais voltadas à hesitação vacinal infantil, identificando os meios utilizados, as abordagens adotadas e os fatores que influenciam as escolhas de pais e cuidadores na tomada de decisão em vacinação.

## MÉTODOS

Trata-se de uma revisão de escopo, conduzida segundo a metodologia do *Joanna Briggs Institute* (JBI) e reportada de acordo com a extensão PRISMA para revisão de escopo (PRISMA-ScR)^18^, de modo a garantir a transparência e o rigor metodológico. Entre os passos seguidos, destacam-se a definição dos critérios de elegibilidade, a seleção criteriosa dos estudos por meio da leitura de títulos, resumos e textos completos, e a extração padronizada dos dados, assegurando consistência e reprodutibilidade ao longo do processo. Na revisão, adotou-se a definição de “infantil” apresentada nos próprios artigos, tendo como fenômeno de interesse a hesitação vacinal nesse grupo etário.

Para a elegibilidade dos estudos, estabeleceram-se os seguintes critérios de inclusão: as pesquisas publicadas entre 2015 e 2023, período definido por suceder à formalização do conceito de hesitação vacinal pelo SAGE, em 2014, em textos disponíveis em inglês, espanhol, português e francês; os estudos que abordassem as estratégias comunicacionais relacionadas à hesitação vacinal infantil; as investigações que explorassem meios, abordagens ou intervenções voltadas à comunicação entre profissionais de saúde, pais ou cuidadores; e as pesquisas empíricas, independentemente do delineamento metodológico. Foram excluídos os estudos de revisão, bem como aqueles que abordavam exclusivamente a vacinação em adultos ou a imunização de forma geral.

As fontes informacionais selecionadas foram: Scopus, *Web of Science*, PubMed, *Scientific Electronic Library Online* (SciELO) e Literatura Latino-Americana e do Caribe em Ciências da Saúde (Lilacs). Para a construção das estratégias de busca, utilizaram-se os descritores DeCS/MeSH: Comunicação em Saúde; Hesitação Vacinal; Recusa de Vacinação; Movimento Contra a Vacinação; Comunicação; Saúde da Criança. Estes foram usados em português para as buscas nas bases SciELO e Lilacs. Nas demais bases, foram utilizados em inglês. Aplicaram-se os operadores booleanos OR e AND para a seleção dos artigos, considerando cada fonte informacional.

A busca nas bases de dados foi realizada em 11 de abril de 2025. Os resultados foram importados para o *software* Rayyan, no qual os textos duplicados foram excluídos. Em seguida, dois pesquisadores realizaram, de forma independente e cega, a triagem por títulos e resumos, acionando um terceiro pesquisador em caso de divergências. Vinte e cinco artigos foram lidos na íntegra e três foram excluídos, por não abordarem estratégias comunicacionais relacionadas à (não)vacinação. Ao final, foram incluídos 22 artigos. O processo de seleção dos estudos está apresentado no diagrama baseado no protocolo PRISMA (Figura).

**Figura:** Diagrama baseado no protocolo PRISMA para revisão de escopo (PRISMA-ScR).

A extração dos dados foi realizada por cinco pesquisadores, de forma independente. As dúvidas ou inconsistências identificadas durante o processo foram discutidas e resolvidas em reuniões coletivas. O processo ocorreu na plataforma Excel e foi orientado pelo referencial das configurações comunicativas no campo da comunicação^19,20^, aplicadas, neste estudo, à comunicação em saúde.

No processo de extração, seguiram-se quatro eixos principais: 1) Caracterização dos artigos, contemplando a identificação dos autores, ano de publicação, título, DOI, periódico, instituição, país e gênero do autor correspondente, utilizado como proxy de gênero. 2) Contextualização dos estudos, incluindo o quadro teórico referenciado, a definição do objeto de estudo, objetivos da pesquisa, localização geográfica (país, estado, região ou *locus*) e grupos ou populações analisadas. 3) Quadro teórico e a delimitação metodológica, englobando o conceito de hesitação vacinal adotado, as mídias utilizadas, as perspectivas dos atores envolvidos (pais/cuidadores/tutores, profissionais de saúde ou gestores) em relação à vacinação e/ou à hesitação vacinal, e as estratégias comunicacionais empregadas nos diferentes meios. Por fim, o quarto eixo refere-se à síntese, na qual foram identificadas as estratégias comunicacionais e seus desfechos.

Para compreender as dinâmicas comunicacionais relacionadas à hesitação vacinal, adotou-se o conceito de configurações comunicativas, definido por Hepp e Hasebrink^20^ como “padrões de processos de entrelaçamento comunicativo que existem ao longo de várias mídias e têm um enquadramento temático que orienta a ação comunicativa” (p. 12).

As configurações comunicativas articulam quatro dimensões interdependentes: (i) formas de comunicação – as modalidades de interação mediada; (ii) conjunto de mídia – as plataformas que sustentam a ação comunicativa; (iii) constelação de atores – os sujeitos que se reconhecem como parte daquela configuração; e (iv) quadro temático – o tema central que dá sentido à interação. Esses elementos são dinâmicos e moldados pelo processo de midiatização, conceito central para analisar a relação entre transformações da mídia, práticas comunicacionais e mudanças socioculturais^19,20^.

A análise dos dados foi orientada pelo referencial teórico das configurações comunicativas, conforme proposto por Hepp e Hasebrink^20^. À luz desse referencial, empregou-se a síntese temática como estratégia de tratamento das informações, apoiada em uma matriz de codificação que possibilitou o agrupamento dos achados em temas centrais.

Esta revisão não foi registrada em plataforma pública, como *International Prospective Register of Systematic Reviews* (Prospero) ou *Open Science Framework* (OSF). Os formulários de extração de dados e a matriz de codificação utilizados na síntese temática estão disponíveis mediante solicitação aos autores.

## RESULTADOS

Foram incluídos nesta revisão 22 artigos. O Quadro 1 sintetiza as principais informações dos estudos analisados. Quanto ao *locus* de realização das pesquisas, a maioria foi realizada nos EUA (n = 9), seguida da Austrália (n = 4) e Itália (n = 2); houve também estudos conduzidos na “internet”, com dados obtidos em inglês. Identificaram-se, ainda, estudos no Brasil, Turquia, Paquistão, Índia, Escócia e Polônia. Os grupos investigados foram: pais e cuidadores, sendo que dois estudos focaram especificamente nas mães; profissionais de saúde (pediatras, médicos de família e enfermeiras responsáveis por vacinação); população em geral; adolescentes e estudantes universitários (de graduação e residência médica).

**Quadro 1:** Síntese da caracterização dos estudos analisados.

Autoria	Objetivo	Desenho metodológico	País, estado, região	Contexto de pesquisa	Grupos/ populações do estudo
Hughes et al.^31^ (2021)	Identificar estratégias retóricas no discurso de hesitação vacinal e elaborar um livro de códigos dos temas antivacinação mais recorrentes, tanto gerais quanto específicos para a covid-19	Qualitativo - análise de conteúdo e produção de *codebook*	EUA	Mídias sociais (Facebook) e *sites* na internet	Pais/cuidadores
Kim et al.^39^ (2022)	Testar a eficácia de mensagens na mudança de atitudes e intenções comportamentais de pais sobre a vacinação contra o HPV	Quantitativo	EUA	Mídias sociais (Facebook) e *sites* na internet	Pais/cuidadores residentes nos EUA
Cole et al.^22^ (2022)	Avaliar o impacto de uma intervenção educativa e de uma ferramenta de comunicação baseada em entrevista motivacional na cobertura vacinal infantil	Quantitativo - intervenção em serviço	EUA	Intervenção que utilizou a estratégia Motive (*Motivational Interviewing Tool to Improve Vaccine Acceptance*)	Profissionais de saúde: pediatria e clínica geral
Duchsherer et al.^30^ (2020)	Analisar como estratégias narrativas entre pais hesitantes ou contrários à vacinação contribuem para a construção de comunidades entre pais hesitantes e que se recusam a vacinar	Qualitativo - análise temática	EUA - estados de: Virginia, West Virginia, Florida, Oregon e Michigan	Depoimentos de pais/cuidadores no website The VaxXed	Pais/cuidadores
Silva et al.^17^ (2023)	Avaliar o efeito de diferentes estratégias de comunicação na intenção de se vacinar e vacinar os filhos, considerando atitudes prévias de hesitação vacinal.	Quantitativo - intervenção comunicacional	Brasil	Ambiente simulado (*online*)	Pessoas maiores de 18 anos residentes no Brasil
Llavona-Ortiz et al.^40^ (2022)	Caracterizar o tipo de informação sobre a vacina contra o HPV visualizada nas redes sociais, o engajamento dos pais com esse conteúdo e sua relação com decisões vacinais.	Quantitativo	EUA	Mídias sociais	Pais/cuidadores
Hopfer et al.^38^ (2019)	Caracterizar padrões de recomendação da vacina HPV entre pediatras, identificando perfis de comunicação associados à hesitação parental.	Quantitativo	EUA	Contexto dos serviços de saúde	Pais/cuidadores
Özen et al.^33^ (2022)	Explorar experiências de médicos de família com pais antivacinas e fornecer dados sobre o movimento antivacinação na Turquia.	Qualitativo - estudo fenomenológico	Turquia - província de Sakarya	Contexto dos serviços de saúde	Profissionais de saúde - médicos de família
Akram et al.^36^ (2021)	Examinar a influência de canais de comunicação, especialmente mídias eletrônicas e sociais, na decisão parental sobre a vacinação contra febre tifoide.	Quantitativo	Paquistão - cidade de Karachi	Diferentes meios de comunicação, incluindo mídias sociais	Pais/cuidadores
Glanternik et al.^23^ (2020)	Avaliar o impacto de uma ferramenta de comunicação para médicos sobre conforto e eficácia percebida ao dialogar com pais hesitantes.	Quantitativo - intervenção em serviço	Internet	Sistema integrado de prestação de cuidados de saúde	Profissionais de saúde - pediatras e médicos de família
Steffens et al.^25^ (2021)	Comparar a eficácia de diferentes estratégias de desmistificação de mitos sobre vacinas na intenção parental de vacinar os filhos.	Quantitativo - intervenção comunicacional	Austrália	Ambiente simulado (online)	Pais/cuidadores
Barton et al.^21^ (2022)	Verificar se o treinamento de residentes em um método de comunicação estruturada influencia comportamentos ao interagir com pais hesitantes.	Quantitativo - intervenção educacional	EUA - Universidade de Louisville	Ambiente simulado (online)	Médicos residentes de pediatria
Randall et al.^34^ (2020)	Identificar estratégias e desafios de comunicação usados por profissionais experientes em conversas com pais altamente hesitantes e propor formas de aprimorar a prática clínica.	Qualitativo - análise temática	Austrália - Melbourne e Sydney	Contexto dos serviços de saúde	Profissionais de saúde
Brunelli et al.^37^ (2021)	Avaliar o alcance de uma campanha de revacinação após falha vacinal e investigar níveis de confiança dos pais em vacinas e fontes de informação.	Quantitativo	Itália - cidade de Codroipo	Contexto dos serviços de saúde	Pais/cuidadores
Panozzo et al.^41^ (2020)	Comparar o impacto de recomendações agrupadas e mensagens personalizadas sobre preocupações maternas na intenção de vacinar contra o HPV.	Quantitativo - ensaio clínico randomizado	EUA	Ambiente simulado (*online*)	Pais/cuidadores
Berry et al.^29^ (2018)	Identificar necessidades de comunicação dos pais sobre imunização para subsidiar o desenvolvimento do sistema de apoio *Sharing Knowledge About Immunisation*/SKAI.	Qualitativo - *grounded theory*	Austrália	Duas grandes cidades e uma de influência regional com alta prevalência de objeção à vacina	Pais/cuidadores
Shay et al.^34^ (2018)	Descrever formas de expressão da hesitação vacinal entre pais, respostas dos profissionais e associações com a vacinação no mesmo dia.	Qualitativo - análise de conteúdo	EUA - Dallas,Texas	Contexto dos serviços de saúde	Pais/cuidadores, adolescentes e profissionais de saúde
Berry et al.^28^ (2017)	Explorar experiências de profissionais de atenção primária em consultas com pais que recusam vacinas, bem como os desafios e estratégias de manejo.	Qualitativo - *grounded theory*	Austrália	Contexto dos serviços de saúde	Profissionais de saúde
Nagar et al.^24^ (2018)	Avaliar a adoção e o impacto do sistema digital Khushi Baby na adesão à vacinação infantil em comunidades rurais.	Quantitativo - intervenção comunitária	Índia	Regiões rurais de Udaipur, Rajasthan	Pais/cuidadores
Pluviano et al.^25^ (2017)	Comparar a eficácia de três estratégias de correção de desinformação sobre vacinas em ambiente experimental controlado.	Estudo de intervenção	Itália - Universidade de Nápolis e Escócia - Universidade de Edimburgo	Ambiente simulado (*online*)	Estudantes destas duas universidades
Lockhart et al.^32^ (2018)	Compreender como uma intervenção de comunicação multifacetada aplicada por equipes médicas pode melhorar as taxas de vacinação contra o HPV.	Qualitativo - avaliação de processo	EUA - Denver, Colorado.	Contexto dos serviços de saúde	Pais/cuidadores e profissionais de saúde
Dolinski et al.^27^ (2023)	Testar a eficácia da técnica “*But You Are Free*” (BYAF) na adesão à vacinação de recém-nascidos.	Delineamento experimental	Polônia	Contexto dos serviços de saúde	Pais/cuidadores
Seytre et al.^42^ (2024)	Desenvolver uma estratégia de comunicação baseada em evidências para aumentar a cobertura vacinal no Níger.	Métodos mistos	Níger	População em geral	População em geral, incluindo adultos responsáveis por crianças

HPV: papilomavírus humano.

Em termos metodológicos, os desenhos de pesquisas foram variados: estudos quantitativos de intervenção^17,21^, incluindo um de delineamento experimental^27^, qualitativos^28^, quantitativos^36^, e um de métodos mistos^42^. Os estudos analisaram diferentes perspectivas sobre a hesitação vacinal infantil. Um conjunto abordou a retórica construída em torno do fenômeno^30,31,33,35^. Outros focaram a comunicação entre pais/cuidadores e/ou profissionais de saúde nas redes sociais^31,39,40^. Também se destacaram estudos sobre a confiança nas vacinas e a influência do movimento antivacina em pais e cuidadores^33,37^.

Diversas pesquisas discutiram estratégias comunicacionais na relação entre profissionais de saúde e pais/cuidadores^17,23,26,29,34^. Outros trabalhos centraram-se nas estratégias comunicacionais voltadas à redução da hesitação vacinal^17,22,24^, além de trabalhos específicos sobre redução da hesitação vacinal^25,26,41^.

A síntese temática realizada levou em conta os quatro aspectos analíticos centrais estabelecidos a partir do referencial teórico de Hepp e Hesebrink^20^, a saber: 1) O conceito de hesitação vacinal; 2) A perspectiva comunicacional empregada: a quem dirige e como se produz; 3) Os tipos de estratégias comunicacionais discutidas; e 4) As estratégias comunicacionais de caráter antivacinal (Quadro 2).

**Quadro 2:** Síntese temática dos achados segundo os quatro aspectos analíticos de Hepp e Hasebrink20.

Aspecto analítico	Temas	Sínteses dos achados
1. Conceito de hesitação vacinal	1.1. Definição clássica (OMS/SAGE)	Predominou a definição da OMS/SAGE, que compreende a hesitação vacinal como atraso ou recusa da vacinação apesar da disponibilidade de serviços^17,21–25,31^.
	1.2. Ampliação conceitual: fenômeno social complexo	Parte dos estudos amplia o conceito, compreendendo a hesitação vacinal como fenômeno social complexo, incluindo a noção de “estado de espírito”, fenômeno em continuum, mecanismos cognitivos e desinformação, além de perspectivas histórico-políticas^21,22,25,30,35^.
	1.3. Ausência de definição explícita	Alguns estudos não apresentam definição formal, associando a hesitação principalmente à desinformação ^26,36,40^, à desconfiança institucional^29,42^ ou a fatores religiosos^33^. Estudos sem definição explícita^27,29,32,38,39,41^.
2. Perspectiva comunicacional: a quem se dirige e como se produz	2.1. Centralidade de pais e cuidadores	A maioria dos estudos dirige-se a pais e cuidadores, especialmente no contexto da hesitação vacinal infantil^24,26–30,32,35–41^. Parte da literatura concentra-se especificamente em pais e cuidadores identificados como hesitantes^29,33,38,41^.
	2.2. Profissionais de saúde como público-alvo	Alguns estudos analisam estratégias voltadas a profissionais de saúde, isoladamente^22,29,33,34^, ou em conjunto com pais/cuidadores^24,28^.
	2.3. População geral e ambientes digitais	Poucos estudos abordam a população geral^17,31,37^ ou analisam redes sociais, websites e plataformas digitais (Facebook/Instagram) como espaços centrais de produção comunicacional^22,31,40^.
3. Tipos de estratégias comunicacionais discutidas	3.1. Mapeamento de narrativas e retórica antivacinal	Estudos que priorizam o mapeamento retórico e a identificação de narrativas antivacinais, com construção de *codebooks* analíticos^31^.
	3.2. Testagem de mensagens e enquadramentos comunicacionais	Testagem do uso de mensagens segundo eixos temáticos e enquadramentos informativos e narrativos^39^. Além de abordagens baseadas na *Effective Communication without Confrontation* centradas na escuta, validação e diálogo^23^.
	3.3. Limites das estratégias corretivas tradicionais	Estratégias baseadas em “mitos versus fatos”, perguntas ou afirmações apresentam resultados limitados ou ausência de diferenças significativas, com indicação de possível efeito rebote^25,26.^
4. Estratégias comunicacionais de caráter antivacinal	4.1. Ecossistemas digitais e disputa de sentidos	As redes sociais configuram ecossistemas heterogêneos, marcados por disputa de sentidos e circulação de narrativas antivacinais baseadas em antagonismo e dano^31^. Comunidades online mobilizam pertencimento identitário, articulando discursos de desconfiança, identidade coletiva e senso de pertencimento a partir deste tema^30^.
	4.2. Infodemia e ampliação da desconfiança	Rumores e infodemia contribuem para a ampliação da desconfiança comunitária e fragilização da confiança institucional^42^.
	4.3. Estratégias de contraponto baseadas em evidências	Mensagens baseadas em evidências científicas aumentam a intenção vacina^39^, e intervenções estruturadas como o Motive podem apoiar profissionais e ampliar a cobertura vacinal^22^.

SAGE: *Strategic Advisory Group of Experts on Immunization*; OMS: Organização Mundial da Saúde.

Quanto ao aspecto referente ao item 1 (conceito de hesitação vacinal), alguns estudos adotaram a definição de 2014 da OMS^4^ que considera a hesitação vacinal como o atraso na aceitação ou a recusa de uma vacina, apesar de sua disponibilidade^17,21,23^. Dentre esses, três estudos definem hesitação vacinal como um fenômeno social complexo, relacionado a crenças sobre saúde em suas dimensões individuais, socioculturais e políticas^21,22,25^. Outros estudos avançam na definição, tratando a hesitação vacinal como um “estado de espírito” permeado por incertezas, conflitos ou oposições^21^, ou como parte de um *continuum* de indecisão^35^, ou ainda como um conjunto de mecanismos cognitivos que favorecem a desinformação^25^. Um estudo apresentou uma perspectiva histórica ampliada para o fenômeno, em que a hesitação vacinal está enraizada em aspectos emocionais, sociais, culturais e políticos que tornam a desinformação “pegajosa” e a crença nas vacinas contraintuitivas^30^.

Vale ressaltar que alguns estudos não apresentaram definição explícita do conceito de hesitação vacinal^27,29,32,35,39,41^, mas o correlacionaram à desinformação^26,36,40^, à desconfiança nas vacinas por seus possíveis efeitos colaterais^29^ ou a motivações religiosas^33^.

Quanto ao público-alvo da comunicação, que diz respeito ao aspecto analítico 2. A perspectiva comunicacional empregada: a quem se dirige e como se produz, 13 estudos tiveram como foco pais e/ou cuidadores^22,24,26,27,29,30,35,36,39^, quatro abordaram especificamente pais/cuidadores hesitantes^29,32^. Oito estudos concentraram-se em profissionais da saúde^21^. Dois estudos consideraram ambos, pais/cuidadores e profissionais da saúde^28,32^e três voltaram-se à população em geral^17,25,31^.

No que diz respeito ao conjunto de mídias utilizadas no enquadramento do tema, quatro estudos destacaram, de forma mais específica, conteúdos produzidos em plataformas sociais como Facebook e Instagram^17,22,31,39^. Apenas dois dos 22 estudos analisaram redes sociais (Facebook) e *websites* como fontes de informação^31,39^. Quanto ao aspecto analítico 3 (tipos de estratégias comunicacionais discutidas), um estudo analisou discursos antivacina a partir de uma abordagem retórica e do contexto de sua produção, resultando na produção de um livro com códigos narrativos recorrentemente utilizados em mensagens antivacinação online, com o intuito de auxiliar diferentes atores no enfrentamento da desinformação durante a campanha de vacinação contra a covid-19^31^. Kim et al.^39^ desenvolveram mensagens estruturadas em cinco eixos temáticos: (1) preocupações com a segurança e os efeitos colaterais das vacinas; (2) desconfiança no sistema de saúde; (3) dúvidas quanto à eficácia da vacina; (4) associações com a atividade sexual; e (5) desinformação sobre o HPV e sua vacina. A estratégia de comunicação “*Effective Communication Without Confrontation*” (ECC) mostrou-se eficaz no estabelecimento de diálogos entre médicos que atendem famílias hesitantes, contribuindo para o estabelecimento de diálogos construtivos^23^.

Outras abordagens comunicativas, como o uso de mitos, perguntas e afirmações, também foram testadas, mas não apresentaram diferenças significativas quanto à intenção de vacinar^39^. Estratégias corretivas do tipo “mito *versus* fato*” podem* ter efeito rebote ao reforçar informações falsas, como a associação entre vacinas e autismo^37^.

No aspecto analítico 4 (estratégias comunicacionais de caráter antivacina), em específico em redes sociais, observou-se atuação ampla e heterogênea, de autoridades governamentais e especialistas à imprensa e ao público em geral^31^. Algumas narrativas constroem antagonistas como um “vilão sombrio” ou apresentam a própria vacina como fonte de dano, sobretudo quanto às vacinações contra a covid-19. Em contraste, intervenções experimentais com pais demonstraram que mensagens baseadas em evidências aumentaram a intenção de vacinar filhos contra o HPV^39^, e que ferramentas como a Motive podem apoiar profissionais de saúde e favorecer a cobertura vacinal^22^.

Estudos revelaram que a exposição a discursos antivacina, especialmente sobre o HPV, pode levar à recusa ou atraso na imunização^40^. A recomendação dessa vacina é influenciada pela percepção parental de risco de antecipação da vida sexual, gerando ambivalência entre profissionais de saúde^38^. Testemunhos de pais no site VaxXed revelam desconfiança nos médicos, autodiagnóstico, defesa da liberdade de escolha e construção de senso de comunidade, expressando a complexa relação entre informação e confiança nas vacinas^30^. Rumores sobre as vacinas contra covid-19, intensificados pela infodemia, também suscitaram desconfiança na comunidade, embora profissionais de saúde mantenham alto grau de confiança^42^.

A construção de vínculos entre médicos e pais/cuidadores mostrou-se essencial para aliviar preocupações e ampliar oportunidades de recomendação vacinal^34^. Em famílias antivacinas, os médicos esclareceram dúvidas sobre efeitos colaterais, origem das vacinas e objeções religiosas. O estudo de Özen et al.^33^ mostrou que os médicos adotaram postura não julgadora, ouviram atentamente os pais e forneceram informações sobre imunizantes e os riscos da não vacinação. Médicos mais experientes relataram utilizar casos clínicos próprios como recurso persuasivo. Embora não impusesse a vacinação, buscaram convencer os pais, alcançando sucesso, apoiados na confiança, comunicação efetiva e compartilhamento de evidências científicas^33^. Intervenções que abordaram preocupações específicas aumentaram a intenção de vacinar^41^.

A comparação entre grupos de pais hesitantes e menos hesitantes revelou que, quanto maior a hesitação vacinal, maior a demanda por transparência e informação^29^. Intervenções específicas, como fichas informativas, imagens de doenças e abordagens presuntivas, mostraram-se positivas na adesão à vacinação contra o HPV, recebendo aprovação de profissionais e pais^32^. Em contrapartida, a técnica BYAF (*But-You-Are-Free Technic*), que pode desencorajar em vez de incentivar a vacinação, deve ser usada com cautela^27^.

## DISCUSSÃO

O enquadramento temático da hesitação vacinal remete ao conceito apresentado pelo modelo conceitual desenvolvido pelo SAGE, voltado a compreender os fatores que influenciam a aceitação ou recusa das vacinas. Ainda assim, os artigos analisados revelam diversidade na forma de compreender o fenômeno; alguns explicitam definições, como a proposta pela OMS, enquanto outros não apresentam conceituação.

As referências da OMS incluem os aspectos comunicacionais entre os fatores que influenciam a hesitação vacinal. Além disso, os documentos destacam que a comunicação é essencial ao sucesso dos programas de imunização, podendo, quando mal empregada, ampliar a hesitação vacinal^4,6^.

A hesitação vacinal envolve incerteza, indecisão, conflito ou oposição à vacinação^11^, indo além da simples recusa ou atraso. Trata-se de um fenômeno que se distribui em um *continuum*, no qual muitos indivíduos não se identificam como “pró-vacina” ou “anti-vacina”, e torna-se particularmente urgente quando se expressa como recusa total ou como atrasos de algumas ou de todas as vacinas^10^.

Klintman^43^ divide a hesitação vacinal em dois tipos: contumaz irracional e contumaz motivada por falta de conhecimento. No primeiro caso, a atitude repousa sobre uma “racionalidade mais arcaica”, ligada a mecanismos de defesa do corpo contra ameaças invisíveis. No segundo caso, o maior e melhor acesso à informação pode fazer uma diferença positiva em segmentos da população cuja hesitação vacinal é moderada ou baixa.

A utilização de definições amplas ou a ausência de clareza conceitual dificulta a comparação de resultados, a operacionalização dos dados e o consenso entre os pesquisadores^5,44,45^. Considerando a complexidade do fenômeno, Couto^46^ defende a necessidade de uma reflexão crítica sobre a visão tradicional, muitas vezes negativa, acerca de indivíduos ou grupos que resistem à vacinação, recomendando uma abordagem que leve em conta múltiplas facetas, incluindo os aspectos sociais e as práticas em saúde.

A recusa à vacinação, distinta da hesitação vacinal, é um fenômeno multifacetado, com motivações diversas^47^. Pais identificados como “recusadores” não formam um grupo homogêneo: podem rejeitar todas as vacinas ou apenas algumas, por preocupações com eficácia, efeitos adversos, desconfiança nas políticas públicas ou na indústria farmacêutica. Como destaca Sobo^47^, há diferença entre recusar por falta de acesso e recusar deliberadamente. Inspirando-se em Marcel Mauss, MacGranahan^48^propõe compreender a recusa como um gesto socialmente denso, enraizado em contextos históricos, éticos e culturais. A recusa em vacinar pode operar como um posicionamento ético que redefine hierarquias e afirma outros modos de viver e cuidar^48^.

A recusa manifesta-se como um posicionamento ativo que rejeita certas aflições e constrói novas formas de sociabilidade. Mais que simples oposição ao Estado ou à ciência, pode representar engajamento moral e político, sustentado por convicções sobre o cuidado responsável com os filhos. Como argumenta McGranahan^48^, a recusa relaciona-se mais à insistência que à resistência: trata-se de uma afirmação de valores e modos alternativos de vida. No estudo de Sobo^47^, a decisão de não vacinar consolida laços sociais entre pais que compartilham uma visão individualizada e crítica da saúde.

Com base nos estudos empíricos analisados e na literatura teórico-conceitual, destaca-se a inexistência de consenso quanto aos usos do termo “hesitação vacinal”, o que pode impactar a formulação de estratégias comunicacionais. Observa-se ainda que a maioria dos estudos se concentra na esfera familiar, sobretudo em pais e cuidadores, tendo as redes sociais como principal mídia de circulação de informações. Compartilha-se, com Hepp e Hasebrink^20^, a compreensão da comunicação como forma de interação social, essencial às relações e construções simbólicas. Nesse sentido, compreender a forma como a comunicação circula e adquire sentido na sociedade torna-se fundamental, o que torna o conceito de midiatização relevante.

O conceito de midiatização descreve como a mídia transforma relações sociais e práticas cotidianas. Duas abordagens principais orientam seu estudo: a institucionalista, centrada na mídia de massa, e a socioconstrutivista, voltada às comunicações diárias e à relação entre evolução midiática e mudanças socioculturais^20^. Hjarvard^49^ propõe que ambas são complementares, definindo a midiatização como o processo pelo qual atividades sociais e culturais passam a ser moldadas e dependentes da mídia – tanto pelo uso que indivíduos e instituições fazem dela quanto pela crescente mediação das interações por plataformas específicas.

Hepp e Hasebrink^20^ propõem o uso do conceito de midiatização para analisar a relação entre transformações na mídia, na comunicação e nas mudanças socioculturais. O foco é compreender como as mídias moldam os processos comunicativos e as interações simbólicas. Nos artigos analisados, destacou-se a presença de conteúdo sobre vacinação em redes como Instagram, Facebook e TikTok, caracterizando o que os autores definem como “comunicação midiática virtualizada”, sustentada por sistemas interativos intencionalmente desenvolvidos.

Os estudos empíricos analisados evidenciaram a formulação de diversos argumentos utilizados para sustentar o discurso antivacina nas redes sociais, com ênfase nas configurações comunicativas envolvidas em contextos diversos de vacinação infantil. Esses achados convergem com os resultados de uma revisão sistemática anterior^50^, centrada na pandemia de covid-19, a qual identificou que crenças em teorias da conspiração, aliadas à desconfiança nos benefícios das vacinas, nas políticas governamentais, nos sistemas de saúde, nos desenvolvedores de vacinas e nos provedores de serviços, além de falta de informação em saúde, contribuem para a disseminação e consolidação de discursos antivacinas. A presente revisão de escopo, no entanto, amplia essa perspectiva ao considerar não apenas a circulação de desinformação, mas também as formas pelas quais diferentes mídias e atores produzem, reproduzem e disputam sentidos sobre a vacinação infantil no campo da comunicação em saúde, elencando, inclusive, abordagens inovadoras e eficazes.

Os resultados indicam que as estratégias comunicacionais mais eficazes são aquelas que priorizam a construção de confiança, o estabelecimento de um diálogo empático e a personalização das mensagens conforme as preocupações específicas de pais e cuidadores. Ferramentas como a Motive e abordagens como a ECC demonstraram potencial para aumentar a aceitação vacinal. Por sua vez, intervenções baseadas em evidências e adaptadas às necessidades dos contextos locais demonstraram potencial para reduzir a hesitação vacinal. Em contrapartida, estratégias informativas no formato “mito *versus* fato” podem ter efeitos contraproducentes, reforçando crenças equivocadas.

Esses achados reforçam a necessidade de integrar o campo da comunicação em saúde às políticas e programas de imunização de forma sistemática. A incorporação de estratégias baseadas em evidências comunicacionais, como o treinamento de profissionais de saúde em escuta qualificada e entrevista motivacional, o uso de mensagens adaptadas aos contextos socioculturais e a construção de redes de confiança entre profissionais, famílias e instituições, pode aprimorar a efetividade das intervenções. Além disso, recomenda-se que diretrizes nacionais e locais de imunização incluam parâmetros específicos sobre comunicação de risco e enfrentamento da desinformação como componentes centrais para a redução da hesitação vacinal.

Entre as limitações dos estudos incluídos na revisão, destaca-se a predominância de estudos exploratórios e de pequeno porte, majoritariamente conduzidos em países de alta renda, o que restringe a discussão dos achados para contextos de média e baixa renda.

Esta revisão de escopo não incluiu avaliação formal do risco de viés, o que restringe a apreciação da robustez dos achados e impede inferências mais firmes sobre a efetividade comparativa das estratégias comunicacionais identificadas. Essa decisão está alinhada às diretrizes do JBI^18^, segundo as quais revisões de escopo não demandam avaliação crítica obrigatória, não excluem estudos com base na qualidade metodológica e visam, sobretudo, mapear a extensão, variedade e natureza das evidências disponíveis, em vez de julgar seu rigor metodológico. Ainda assim, à medida que a literatura empírica sobre o tema avance, recomenda-se que estudos futuros, especialmente aqueles voltados a avaliar a eficácia de estratégias comunicacionais para enfrentar a hesitação vacinal, incorporem avaliação sistemática do risco de viés, de modo a fortalecer a interpretação dos efeitos e a comparabilidade entre intervenções.

## CONCLUSÃO

Os resultados indicam que as mídias exercem papel ambivalente na comunicação sobre vacinas: de um lado, veiculam desinformação e reforçam crenças equivocadas, de outro, podem difundir mensagens pró-vacinação baseadas em evidências e sensíveis ao contexto sociocultural. Linguagem clara e acessível, uso de ferramentas como a Motive e análise de discursos antivacina podem ampliar a intenção de vacinar. Contudo, a comunicação não confrontacional, embora fortaleça a relação médico-paciente, não garante, isoladamente, maior cobertura vacinal.

Diante desses achados, conclui-se que o enfrentamento da hesitação vacinal requer estratégias integradas que articulem políticas públicas orientadas por evidências e ações de comunicação em saúde personalizadas e sensíveis às especificidades socioculturais dos públicos. O fortalecimento de abordagens comunicacionais baseadas em evidências, aliadas a políticas de imunização socialmente inclusivas, pode subsidiar práticas profissionais e a formulação de diretrizes capazes de promover maior confiança nas vacinas e adesão sustentada aos programas de imunização infantil.

## Data Availability

Os dados estão disponíveis mediante solicitação ao autor correspondente.
